# Biased Recognition of Facial Affect in Patients with Major Depressive Disorder Reflects Clinical State

**DOI:** 10.1371/journal.pone.0129863

**Published:** 2015-06-03

**Authors:** Paula Münkler, Marcus Rothkirch, Yasmin Dalati, Katharina Schmack, Philipp Sterzer

**Affiliations:** Visual Perception Laboratory, Department of Psychiatry and Psychotherapy, Campus Charité Mitte, Charité—Universitätsmedizin Berlin, Berlin, Germany; Bournemouth University, UNITED KINGDOM

## Abstract

Cognitive theories of depression posit that perception is negatively biased in depressive disorder. Previous studies have provided empirical evidence for this notion, but left open the question whether the negative perceptual bias reflects a stable trait or the current depressive state. Here we investigated the stability of negatively biased perception over time. Emotion perception was examined in patients with major depressive disorder (MDD) and healthy control participants in two experiments. In the first experiment subjective biases in the recognition of facial emotional expressions were assessed. Participants were presented with faces that were morphed between sad and neutral and happy expressions and had to decide whether the face was sad or happy. The second experiment assessed automatic emotion processing by measuring the potency of emotional faces to gain access to awareness using interocular suppression. A follow-up investigation using the same tests was performed three months later. In the emotion recognition task, patients with major depression showed a shift in the criterion for the differentiation between sad and happy faces: In comparison to healthy controls, patients with MDD required a greater intensity of the happy expression to recognize a face as happy. After three months, this negative perceptual bias was reduced in comparison to the control group. The reduction in negative perceptual bias correlated with the reduction of depressive symptoms. In contrast to previous work, we found no evidence for preferential access to awareness of sad vs. happy faces. Taken together, our results indicate that MDD-related perceptual biases in emotion recognition reflect the current clinical state rather than a stable depressive trait.

## Introduction

Current concepts of depression are largely based on cognitive theories [1] according to which depression is characterized by a negative bias in perception. In line with this notion, there is substantial empirical evidence showing that perception in patients with major depressive disorder (MDD) is characterized by blunted responsiveness to emotionally positive information as well as an increased tendency to perceive emotionally neutral visual information as negative [2–7].

A critical point for the understanding of the etiological and developmental aspects of MDD is the question whether such a negative bias represents a stable vulnerability factor which persists beyond a depressive episode. In this case the trait-like characteristic of a negative bias could prove useful for the identification of persons at risk [8,9]. If, in contrast, a negative bias is confined to the depressive episode, it could serve as a state marker for MDD, for instance to objectively monitor treatment responses [10]. Previous research has yielded heterogeneous results regarding temporal stability of such a negative perceptual bias. In some studies biased emotion recognition was observed even after recovery from major depressive episodes [11,12], while other studies reported a reduced negative perceptual bias and improved emotion discrimination after symptom remission [13–16].

Two important factors may primarily account for the inconsistencies between previous studies. Firstly, the ability to differentiate between state and trait markers of MDD crucially depends on the experimental design. In a number of previous studies perceptual biases in remitted MDD patients were compared to never-depressed healthy controls [11–13,15,16]. A more direct assessment of the temporal stability of perceptual biases would be provided by a repeated-measures design in which patients with MDD are tested during a depressive episode and after remission. With this approach, the development of a perceptual bias can be directly related to the development of depressive symptomatology. A second important factor is the stimulus material used to probe biased perceptual processing of emotional information in MDD. In several previous studies, participants were exposed to face stimuli displaying emotional expressions at full intensities [12,16], to schematic faces [14] or to drawings of facial expression [15]. Misclassifications of emotional expressions of such stimuli may lack the sensitivity to capture altered emotion processing in MDD. Greater sensitivity can be achieved by varying the intensity of the facial expression including rather subtle changes in emotional expressions. Recognition of such subtle expressions is more closely related to emotion recognition in everyday life, since emotions displayed by others are usually less intense than in standard face stimulus sets. The use of morphed emotional faces can yield relevant information on the nature of biased emotion perception along a particular dimension (e.g. for the transition from happy to sad expressions). Moreover, such an approach can also help to differentiate whether impairments in emotion recognition are due to misclassification of ambiguous expressions, that is, a shift in categorical emotion recognition [5,17], or rather a general uncertainty in emotion recognition. The latter would be reflected by a flattened response pattern. To the best of our knowledge, the stability of the MDD-related perceptual bias in the recognition of subtle changes in emotional face expressions has not yet been tested in a repeated-measures design.

In the current study we investigated the temporal stability of negative perceptual bias in patients with MDD. We used a repeated-measures design, in which patients were tested during a depressive episode (T1) and three months later (T2). In a forced-choice task, participants were asked to indicate the valence of expressions of face stimuli that varied with respect to their degree of expressed happiness or sadness. In line with previous research, we expected biased emotion recognition in patients with MDD in comparison to healthy participants at T1 [5]. For the comparison between the two time points, we hypothesized that an unchanged perceptual bias at T2 would represent a stable trait marker of MDD, whereas a reduction in negative perceptual bias from T1 to T2 would argue for a state marker.

A second focus of our study was the question whether a negative perceptual bias in emotion recognition may be related to previously reported biases in automatic emotion processing [18]. In line with this notion we recently found that faces with sad expressions had privileged access to awareness compared to happy faces in patients with MDD, indicating an automatic bias towards negative emotional stimuli in MDD [19]. In the present study we therefore included a second task that probed the effects of emotional expressions on the potency of face stimuli to gain access to awareness. The purpose of this task was to investigate whether a negative bias in the recognition of morphed emotional expressions—reflecting the conscious evaluation of the stimuli—would correlate with preferential access of negative emotional information to awareness—reflecting automatic stages of visual information processing.

## Materials and Methods

### Participants

31 patients with MDD and 28 healthy control participants matched for age, gender and educational status were tested. We included patients diagnosed by a trained psychiatrist as having moderate or severe MDD according to DSM-IV criteria. Diagnoses were made based on the Hamilton Depression Rating Scale (HAMD) [20] and Beck’s Depression Inventory (BDI) [21] performed by the treating physician. Eleven patients were inpatients at the Department for Psychiatry and Psychotherapy at Charité —Universitätsmedizin Berlin, Campus Charité Mitte (Berlin, Germany). Eleven patients were under treatment in the day-clinic and three were treated as outpatients of the same department. The remaining six patients were outpatients recruited through internet advertisement. Control participants were recruited through internet advertisement or the department’s volunteer data base. All participants had normal or corrected-to-normal vision. None of the participants had a history of brain injury, neurological disorders, or current substance abuse. The Structured Clinical Interview for the DSM-IV (SCID) was used by specially trained medical students to screen for psychiatric illnesses [22]. None of the patients with MDD had any psychiatric comorbidity according to DSM-IV axis I [22] except for anxiety disorder. There was no evidence of past or present psychiatric disorders in any of the control participants. Severity of depression was assessed with the Hamilton Depression Rating Scale (HAMD) [20] and the Beck’s Depression Inventory (BDI) [21] by trained medical students on the day of testing. Patients were included in the study if they had a HAMD score of 18 points or higher. According to these criteria excluding patients with above mentioned neurological or psychiatric history, the final sample sizes comprised 26 patients with MDD and 28 healthy participants.

Patients with MDD and healthy controls performed the experiments at two time points: The first measurement was performed when patients were acutely depressed (T1). A follow-up measurement (T2) was scheduled for three months later. Control participants were re-tested after the same time interval of three months. Five patients and four healthy controls who took part in the experiment at T1 could not be contacted for T2. Therefore, repeated-measures analyses including T1 and T2 were based on a final sample of 21 patients with MDD and 24 healthy participants. To estimate intelligence level, we assessed total years of training and highest educational achievement on a six-point-scale reflecting the three possible school degrees in Germany and the subsequent professional training (1 = nine-year school degree; 2 = nine-year school degree plus apprenticeship; 3 = 10-year-school-degree; 4 = ten-year school degree plus apprenticeship; 5 = 12 or 13-year-degree; 6 = 12 or 13-year-degree plus university degree). Additionally, all participants performed the WST (Wortschatztest), a verbal intelligence test [23]. Handedness was assessed using the Edinburgh Handedness Inventory [24].

At T1, seven patients were tested before antidepressant treatment was started; 19 were under antidepressant medication as follows: Selective serotonin reuptake inhibitors (8 patients), bupropion (6), venlafaxine (5), mirtazapine (2), tricyclic antidepressants (2), atypical antipsychotics (4), anticonvulsants (4) and benzodiazepines (3; 2 not paused before testing). Thirteen patients received combined pharmacological treatment, six received one single drug. For one patient (not included in T2) data on medication are missing. Patients under regular treatment with benzodiazepines were only included when they reported no drowsiness. One patient underwent electroconvulsive therapy (ECT) that had started one week before testing. At T2, medication was as follows: serotonin reuptake inhibitors (7 patients), bupropion (6), venlafaxine (4), tricyclic antidepressants (2), atypical antipsychotics (3), anticonvulsants (2) and lithium (3). Four patients received no antidepressants, seven patients received combined pharmacological treatment, and ten received one single drug. Among the four patients who did not receive treatment at T2, two had stopped taking antidepressant drugs between the two time points of testing and the two remaining had not received any pharmacological treatment at all. Except for the latter two patients, all patients tested at both time points underwent pharmacological treatment.

### Ethics statement

All patients and control participants gave informed and written consent prior to their participation in the study, which was approved by the local ethics committee.

### Stimuli

Both experiments were performed in a dimly lit room with sound absorption. Participants were seated in front of a 19 inch Samsung CRT monitor (resolution: 1024 x 768; frame rate: 60 Hz). A stable effective viewing distance of 50 cm was secured by a chin-and-head-rest front-mounted to a custom-made mirror stereoscope. For stimulus presentation we used MATLAB (The MathWorks, USA) with the Cogent 2000 toolbox (http://www.vislab.ucl.ac.uk/cogent.php), running on a Pentium 4 computer.

### Morphing technique in Experiment 1

**Fig 1:**
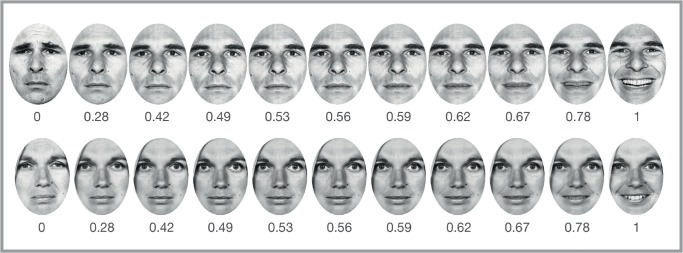
Examples of the morph steps for two face exemplars. Numbers indicate proportion of happiness expressed by the face: 0 corresponds to 100% sad, 1 to 100% happy, and 0.5 to 100% neutral. The middle position (0.56) is a slightly happy expression of 12% (see text).

Stimuli were pictures of four female and four male actors from the Ekman and Friesen series “Pictures of Facial Affect” (http://www.paulekman.com) displaying neutral, happy, and sad expressions. Each of the faces underwent a morphing process using FantaMorph (version 4.1, January 2009 [http://www.fantamorph.com]). The morphed pictures were generated using three genuine photographs of faces with a sad, a happy, and a neutral emotional expression. We used each respective neutral picture as a starting point and morphed it towards either happiness or sadness in five steps, resulting in a set of 11 pictures for each face identity. Pilot tests were performed with 15 healthy participants to determine adequate morph steps. We found that 10% increments, as used in previous studies (e.g.[25], resulted in a steep switch from sad to happy judgments. To maximize sensitivity in the near-neutral range, we therefore used morphing steps that were based on a logarithmic scale (Fig 1). In our pilot experiments, we observed that faces with a neutral expression had a slight tendency to be judged as sad rather than happy. In contrast, faces containing a proportion of 12% of the happy expression were equally often judged as being happy and sad across participants. We thus defined these faces as the neutral midpoint between the two emotional expressions.

### Procedure

#### Experiment 1: Emotion recognition

Each trial began with the presentation of a face on a grey background in the center of the screen. After 1000 ms the face was removed from the screen. Participants were instructed to indicate via key press whether they judged the face to display a happy or a sad expression. They were informed that there might be ambiguous expressions, but that they should always decide whether it was a rather happy or rather sad face. Right and left arrow keys on the keyboard were marked with a happy and a sad schematic face, respectively. Participants were instructed to press the button as soon as they had made a decision, but without time pressure. After the participant had pressed a key and an additional inter-stimulus-interval of 2000 ms, the next trial followed. If no key was pressed, the next trial followed after 20 s. Each of the eleven morph increments was presented 16 times, i.e., each face identity twice in one morph increment which amounts to a total of 176 trials. Participants were excluded if their hit rate was below 75% in one of the 100% conditions. Four control participants and none of the depressed patients were excluded according to this criterion, resulting in a final sample of n = 26 patients and n = 24 healthy control participants at T1. For T2, this exclusion criterion led to a final sample size of n = 21 patients and n = 20 control participants.

#### Experiment 2: Access to awareness

The experimental design of experiment 2 largely resembled the design of a previous study by [19]. It was originally based on a behavioral study in healthy volunteers [26]. Stimuli were displayed on a grey background. During the experiment, two white-line squares (8.5° x 8.5°) were presented side by side on the screen and were viewed through the mirror stereoscope such that only one square was visible to each eye. In the center of each square a white fixation cross (0.5° x 0.5°) was displayed. Participants were asked to maintain stable fixation during the experiment. The experiment comprised a continuous-flash-suppression (CFS) condition, in which high-contrast Mondrian-like pattern masks [27,28] measuring 8.3° x 8.3° were flashed to one eye at a frequency of 10 Hz, while a face stimulus (2.5° x 3.6°) was faded-in to the other eye in one of the four quadrants of the display. The contrast of the face stimulus was ramped up slowly from 0% to 100% within a period of 2 s to ensure invisibility of the face at the beginning of each trial, and then remained constant until the participant made a response on a computer keyboard (keys F, J, V and N) indicating the face’s location. The high-contrast Mondrian-like pattern mask was faded out by linearly decreasing its contrast from 2 s until the end of each trial [29] unlike the study by [19] where the mask was stopped abruptly at the end of each trial. Observers were instructed to respond as fast and as accurately as possible as soon as any part of the face became visible. Yet another difference to the study by [19] was that no fearful faces were included, as no effect had been shown for fearful faces in this earlier study. In addition to CFS, a control condition was used that did not involve binocular rivalry. Control trials started with the presentation of only a flashing Mondrian pattern to one eye. A face stimulus was then shown in one of the four possible locations as in the CFS condition, but with full contrast and to both eyes at a random time between 2 and 8 s after trial onset. Note that the control condition was not designed to match the CFS condition perceptually, but merely to control for possible systematic between-group differences in reaction times to the appearance of faces with different emotional expressions. Both CFS and control trials ended after the participant’s key press. CFS trials were discarded if no key was pressed within 10 s [29]. The inter-trial interval was 2 s. The experiment started with a short training block. The whole experiment comprised 216 trials (144 CFS and 72 control trials) split up into six blocks. CFS and control trials were intermixed randomly within each block. For a more detailed description be referred to [19].

### Data analysis

#### Sample characteristics

In order to test for between-group differences in age and verbal intelligence, two-sample t-tests were performed. Chi-square-test was used to probe differences in the proportion of male and female participants and Mann-Whitney U-tests for differences in years of education, training degree and score on the Edinburgh Handedness Inventory (EHI).

#### Experiment 1: Model selection

The following models were fitted to each individual’s responses to identify the model providing the best fit to the observed behavior. For all models, participants’ behavior was operationalized as the proportion of ‘happy’ responses for each respective morph step. Firstly, logistic functions of the following form were fitted to each participant’s individual manual responses on the basis of a non-linear least squares approach:

$$f(x)=\frac{1}{1+e^{a(b-x)}}$$ (1)

In this equation, *a* denotes the slope of the function and *b* denotes the point of subjective equality (PSE), that is, the location of the function on the continuum of the morphed faces from fully expressed sadness to happiness. The two parameters allow for a specific characterization of participants’ response profiles: The steepness of the slope (*a*) indicates participants’ behavior in the transition from sad to happy faces. A higher value for slope reflects a more abrupt switch from sad to happy. The PSE (*b*) indicates the location of the inflection point of the fitted function, that is, the morphing step at which faces are equally often judged as happy and as sad. It thus reflects each participant’s criterion for the discrimination between happy vs. sad expressions. Hence, the greater the PSE, the greater the proportion of the happy expression that the individual needs to judge the face as being happy.

Secondly, we fitted logistic functions with four parameters to participants’ behavioral responses [30]. In addition to the two-parameter model explained above, the lower (*c*) and upper asymptotes (*d*) of the logistic curves are included in the model as free parameters:

$$f(x)=d+\frac{c-d}{1+{\left(\frac{x}{b}\right)}^{a}}$$ (2)

Thirdly, we performed linear regressions between the logit of participants’ responses and the corresponding morph steps with *β*_*0*_ being the intercept and *β*_*1*_ being the slope of the regression line:

$$f(x)=\beta_{0}+\beta_{1}*\log(\frac{x}{1-x})$$ (3)

**Fig 2:**
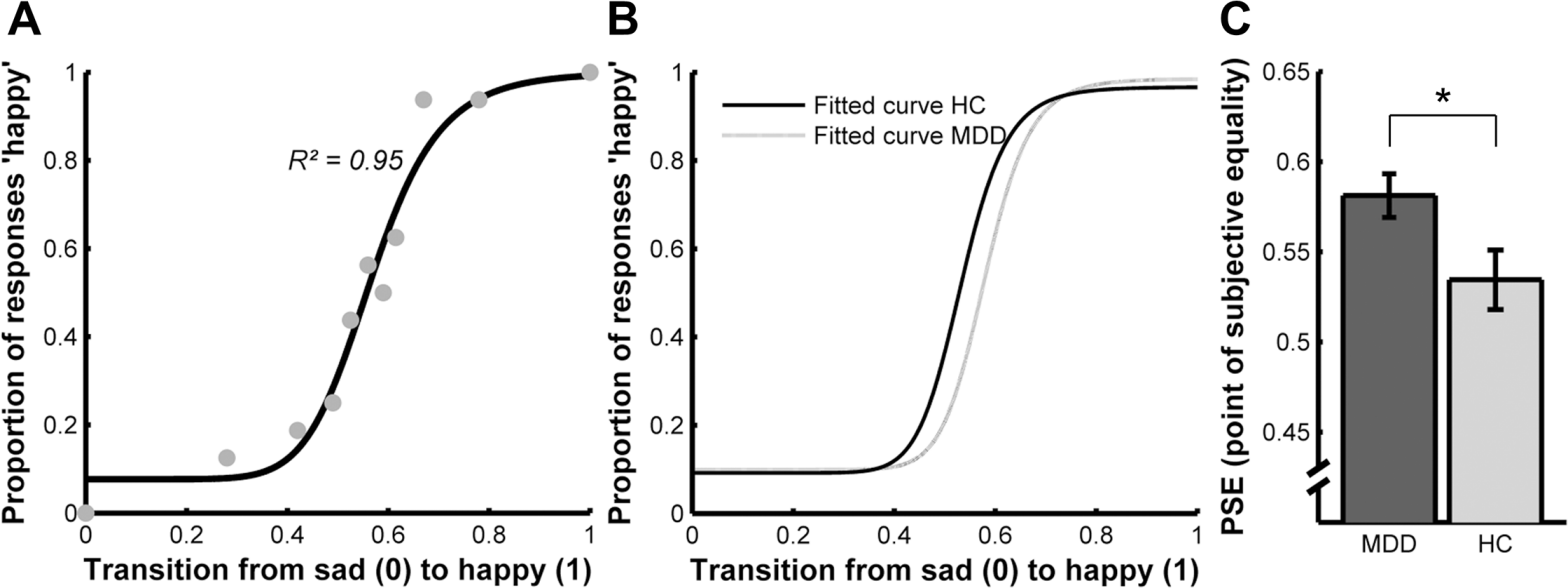
Negative perceptual bias in patients with MDD. Patients with MDD recognized facial expression as happy if a higher proportion of happiness is expressed by the face compared to HC. MDD = major depressive disorder; HC = healthy controls **(A):** Example of a fitted logistic function to the behavioral responses for one representative participant. Intensity of the affective expression is displayed on the y-axis. Transition from sad to happy corresponds to values between 0 and 1 on the x-axis. 0 corresponds to the fully sad expression, 1 is attributed to a happy expression. A y-value of 1 corresponds to a classification of the face as happy in each trial and a y-value of 0 to the response ‘sad’ in each trial, 0.5 is assigned if a face is equally often classified as happy and sad. The x-value indicates the PSE of the curve corresponding to the criterion for the categorical shift. The categorical shift from sad to happy indicates the morphed facial expression that is equally often categorized as happy and sad. **(B):** Bar plot, displaying the mean PSE at T1 for both groups. *p = 0.025. Error bars denote within-subject standard errors of mean [53].

To identify the most appropriate model, the goodness of fit of each model was assessed on the basis of *R²* values adjusted for degrees of freedom, that is, for the number of parameters of the respective model. To this end, individual *R²* values were transformed to *z*-values using Fisher’s z transformation, averaged across all participants and time points, and finally transformed back to *R²* values. According to this procedure, the linear regression model (Eq (3), *R²* = 0.844) was clearly outperformed by the logistic models, for which the four-parameter model (Eq (2), *R² =* 0.949; Fig 2A) provided a slightly better fit than the logistic model comprising two parameters (Eq (1), *R² =* 0.945). All subsequent analyses were thus performed on the parameters estimated on the basis of the four-parameter logistic model. Importantly, single-subject goodness of fit values for this model did not differ between groups (two sample t-test: t(48) = 0.64; p = 0.52) or time points (paired-sample t-test: t(40) = 1.32, p = 0.19).

#### Experiment 1: Statistical analysis of model parameters

Firstly, we analyzed group differences for slope and PSE at T1. To this end, two-sample t-tests were performed for slope and PSE separately. Secondly, we subjected slope and PSE values to 2 x 2 repeated-measures ANOVAs with factors group and time (T1, T2).

Moreover, for MDD patients we performed a Pearson correlation to test whether a reduction in depressive symptoms from T1 to T2, indicated by changes in BDI score, is related to an improvement in emotion recognition from T1 to T2, indicated by changes in the PSE of the logistic function.

#### Experiment 1: Mixed-effects models

The two-level approach described above, comprising the estimation of function parameters on the first level and statistical inference of these parameters on the second level, does not take into account the correlation between function parameters. This approach can thus produce spurious findings, especially for categorical outcomes [31]. We therefore performed additional corroborative analyses to overcome these shortcomings by modelling our data in the framework of a mixed-effects approach, in the context of which fixed effects are estimated separately from random effects [32]. In order to model psychophysical data, generalized linear mixed models (GLMMs) have been proposed allowing for the specification of the relation between predictor and outcome variables [33]. To fit GLMMs to our data we used the lme4 package provided for the statistical software R [34]. For data analysis at T1, the model had the following structure:

$$Y=\beta_{0}+\beta_{1}X_{1}+\beta_{2}X_{2}+\beta_{3}(X_{1}X_{2})+bZ$$ (4)

In this equation *Y* denotes the binomial response of each participant in each single trial such that Y = 0 for trials in which a face was judged as being sad and Y = 1 for trials in which a face was judged as expressing happiness. *X*_*1*_ and *X*_*2*_ are the fixed-effects parameters morph level (i.e. proportion of happiness) and group, respectively. *X*_*1*_ *X*_*2*_ represents the interaction between the two fixed-effects parameters and *β*_*1*_ to *β*_*3*_ are the fixed-effects coefficients. Subjects were included in the model as random effect, denoted by *Z* and the coefficient *b*. For the analysis of the data at T1 and T2, time point (*X*_*3*_) was added as a fixed effect to the model in Eq (4) as well as the interactions of time point with the other fixed effects:

$$\begin{array}{l} Y=\beta_{0}+\beta_{1}X_{1}+\beta_{2}X_{2}+\beta_{3}X_{3}+\beta_{4}(X_{1}X_{2})+\\ \beta_{5}(X_{2}X_{3})+\beta_{6}(X_{1}X_{2}X_{3})+bZ \end{array}$$ (5)

#### Experiment 2

For each trial, suppression times in the CFS condition and reaction times in the control condition were defined as the interval from face presentation onset until participant’s button press. Only trials with correct responses were included in the analyses. For each participant, outlier responses, defined as response times beyond 1.5 times the inter-quartile-interval below the first quartile or above the third quartile [35] were discarded. The proportion of outliers was low, 2.5% in the patient group and 1.3% in the control group. Mean response times were calculated for each emotion in the CFS-condition and control condition, respectively. Emotion-specific mean reaction times in the control condition were subtracted from the respective mean suppression time in the CFS condition to control for possible systematic reaction time differences. To reduce the influence of between-subject differences in overall suppression time and thereby increase sensitivity for within-subject differences, we analyzed the suppression time for happy and sad expression in relation to the neutral expression [19]. For statistical analysis we performed a repeated-measures ANOVA with the between-subject factor group and the within-subject factor emotion. One healthy control participant had to be excluded from analysis at T2 due to reaction times of more than 4 seconds in the control condition which was designed as a task controlling for reaction time.

## Results

### Sample characteristics

There were no differences in the demographic variables age, gender, years of education, training degree, intelligence and handedness (see Table 1). Scores of BDI and HAMD were significantly higher in the patient group compared to the healthy control group (two-sample t-tests, p<0.001). A significant reduction of BDI and HAMD scores in the patient group after three months was observed (paired t-tests, p<0.001). None of the differences in the demographic variables became significant after exclusion of participants as stated in the Data analysis section. For participants included at time point T2, the difference in years of education between the two groups approached significance (p = 0.053).

**Table 1:** Demographic data.

	MDD	HC	p-value	Data analysis
**Sample size**				
T1	26	28		
T2	21	24		
**Age in years (mean ± SE)**				
T1	39.3 (±2.48)	40.2 (±2.28)	0.806	Two-sample t-test
T2	40.0 (±2.86)	40.0 (±2.44)	0.970	
**Sex (male/female)**				
T1	11/15	13/15	0.761	Chi²-test
T2	10/11	12/12	0.873	
**Education in years (median)**				
T1	15	13.5	0.156	Mann-Whitney U-test
T2	16	14	0.053	
**Highest training degree (median)**				
T1	5	4	0.444	Mann-Whitney U-test
T2	5	4	0.247	
**Verbal intelligence in IQ (mean ± SE)**				
T1	107.7 (±0.33)	105.3 (±2.01)	0.393	Two-sample t-test
T2	109.6 (±2.33)	105.1 (±1.78)	0.154	
**EHI (median)**				
T1	100	100	0.843	Mann-Whitney U-test
T2	90	73	0.980	
**BDI (mean ± SE)**				
T1	34.2 (±1.64)	2.8 (±0.55)	<0.001	Two-sample t-test
T2	22.8 (±2.64)	2.4 (±0.58)	<0.001	
**HAMD (mean ± SE)**				
T1	25.8 (±1.05)	1.25 (±0.32)	<0.001	Two-sample t-test
T2	15.1 (±1.47)	1.0 (±0.26)	<0.001	

*IQ = intelligence quotient*, *assessed with WST (Wortschatztest); EHI = Edinburgh Handedness Inventory; BDI = Beck‘s Depression Inventory; HAMD = Hamilton Rating Scale for Depression; SE = standard error of the mean*

### Experiment 1

#### MDD is associated with negative perceptual bias

Logistic functions were fitted to each participant’s responses (Fig 2A). Mean slope and PSE were calculated for each group.

For the data assessed at T1, two-sample t-tests yielded a difference in PSE between the two groups with higher PSEs for patients with MDD (Fig 2B and 2C), but no significant difference in slope (t(48) = 0.591; p = 0.558).

A further test of participants’ responses at T1 was provided by modelling the data using GLMMs (Eq (4)). This analysis yielded a significant main effect of group (β_2_ = -1.54, z = -4.55, p < 0.001) and, most importantly, a significant interaction between group and the morph step of the face (β_3_ = 2.26, z = 4.74, p < 0.001). This indicates that the judgments about the emotional expressions of the faces differed between groups and that this difference was dependent on the proportion of happiness expressed by the faces.

#### Perceptual bias is affected by change in depressive symptoms

The analysis including the second measurement was restricted to participants who had taken part in the experiment both at T1 and T2. The control group therefore amounts to n = 20 and the patient group to n = 21.

**Fig 3:**
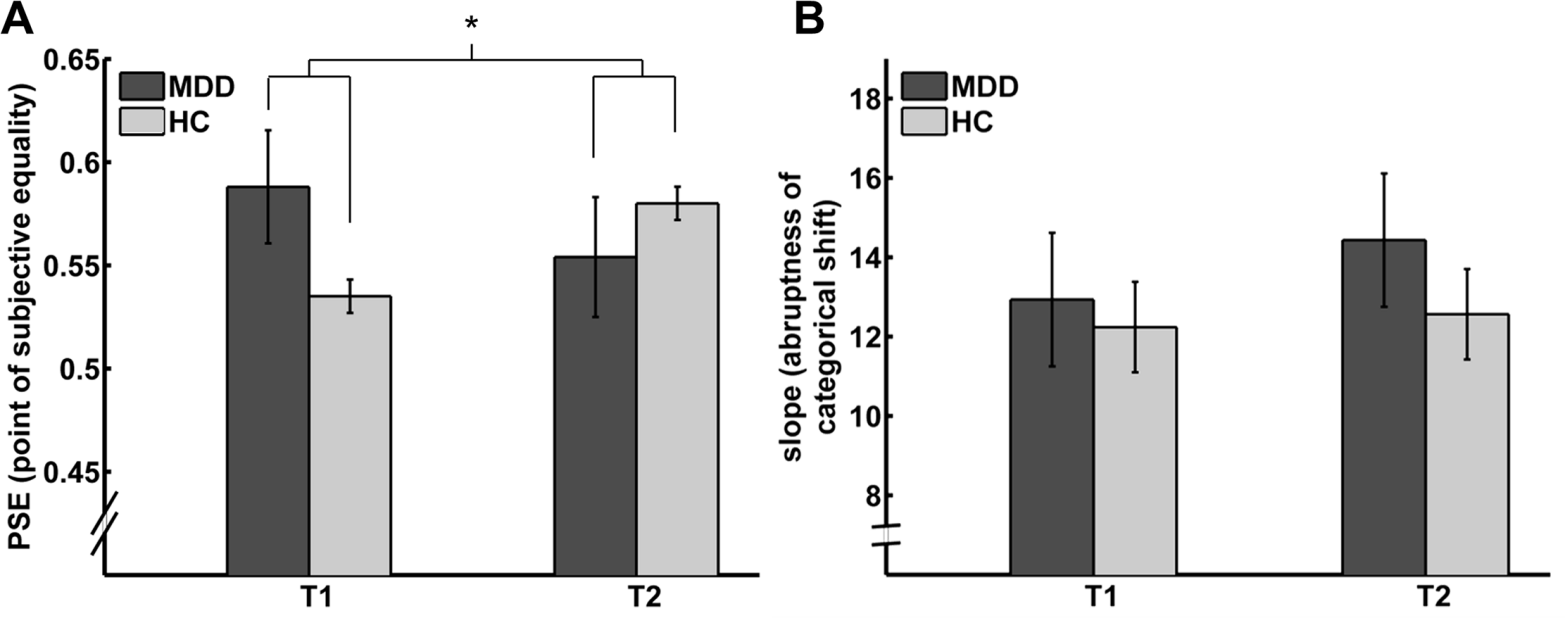
Perceptual bias is reduced in patients with MDD with diminished depressive symptoms. MDD = major depressive disorder; HC = healthy controls. Bar plots depicting mean PSEs **(A)** and slopes **(B)** of the logistic functions for the two groups and two time points. A significant interaction of time and group was observed for the PSE, indicating the inflection point of the logistic functions (p < 0.001). For the slopes of the logistic functions, indicating the abruptness of the categorical shift from sad to happy, no main effects or interactions were found. *: interaction of time and group, p < 0.001. Error bars denote within-subject standard errors of mean [53].

With regard to the PSE of the logistic function, the inflection point where faces were equally often categorized as either happy or sad, a 2 x 2 factorial repeated measures ANOVA with the within-subject factor time and the between-subject factor group showed neither a main effect of group (F(1,39) = 0.433; p = 0.515) nor a main effect of time (F(1,39) = 0.114; p = 0.738). However, this analysis revealed a significant interaction of time x group (F(1,39) = 12.721; p < 0.001; Fig 3A). Post-hoc paired t-tests within each group showed a significant reduction of the PSE in the MDD group (t(20) = -2.362, p = 0.028) and an increase in the control group (t(19) = 2.669, p = 0.015). In order to examine the sensitivity of the statistical analysis, we additionally performed a post-hoc power analysis for the control group [36]. This analysis yielded a statistical power of P = 1 - β = 0.757 and an effect size of d = 0.626, which approximately corresponds to the commonly recommended statistical power of P = 0.8 [37]. Furthermore, we analyzed whether the observed time-related difference in the control group was caused by statistical outliers. Outliers were neither detected at time point T1 nor at T2. However, there was one outlier regarding the difference in PSE between T1 and T2. The interaction of time x group (F(1,38) = 11.233, p = 0.002) as well as the increase in the control group (t(18) = 2.409, p = 0.027) remained significant after outlier exclusion. Together, this indicates a low probability that the observed effect in the control group reflects a false positive finding. For the slope of the logistic functions, a two-factorial ANOVA with repeated measures showed neither a main effect of group (F(1,39) = 0.287; p = 0.595), nor a main effect for time (F(1,39) = 0.198; p = 0.659), nor an interaction of time and group (F(1,39) = 0.082; p = 0.776; Fig 3B).

The effect of time was confirmed by the mixed-effects analysis (Eq (5)). This analysis revealed a significant interaction between group and time (β_5_ = 2.10, z = 5.04, p < 0.001) as well as a significant three-way interaction between group, time, and morph level of the faces (β_6_ = -2.74, z = -3.74, p < 0.001).

**Fig 4:**
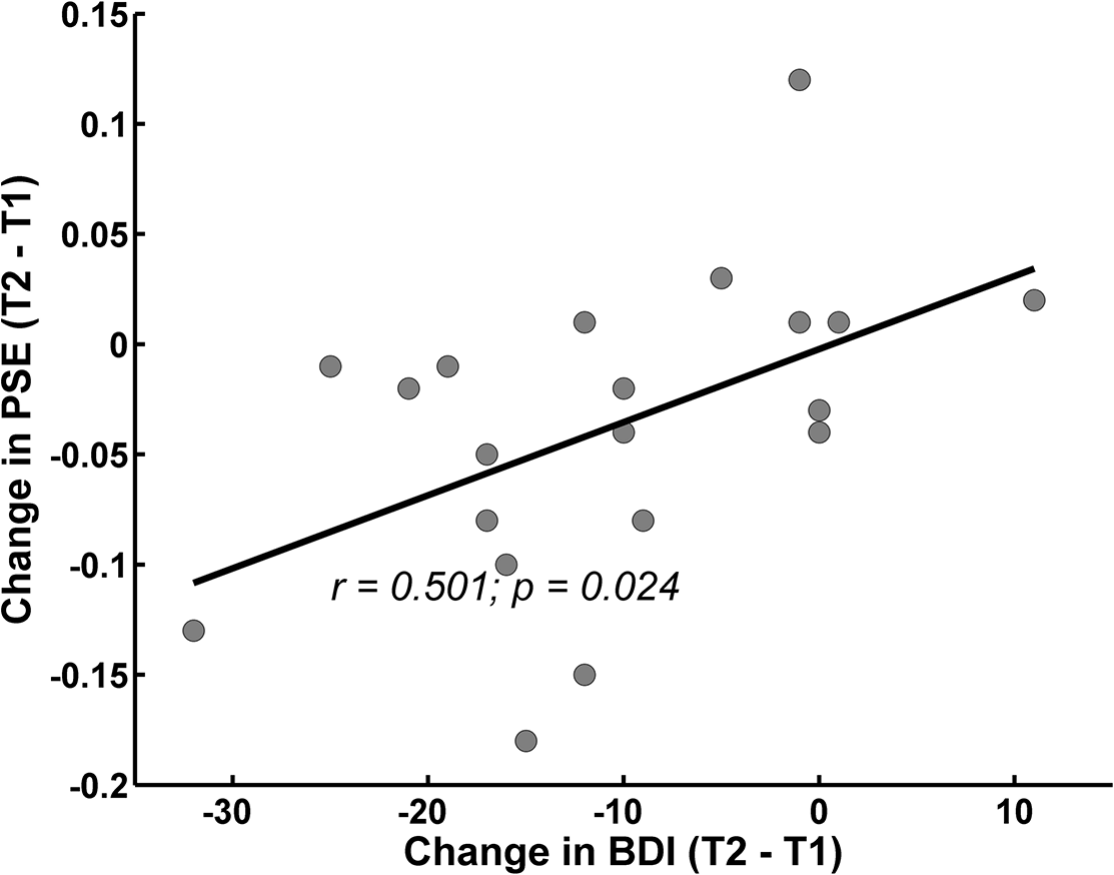
Correlation between change of depressive symptoms and change of perceptual bias in patients with MDD. For patients diagnosed with depression, the change in the degree of severity of depressive symptoms between the two time points of testing correlated positively with the change of the perceptual biases between the two sessions. The severity of depressive symptoms is indexed by BDI scores. The perceptual bias is indicated by the PSE of the individual logistic functions.

To investigate whether reduced depression severity from T1 to T2 was related to the observed shift in perceptual bias in depressed patients, a Pearson correlation between the change in BDI and change of the PSE was performed. This analysis yielded a positive correlation (r = 0.501; p = 0.024; see Fig 4) indicating that a greater change in perceptual bias was associated with a greater reduction of depressive symptoms from T1 to T2.

### Experiment 2

**Fig 5:**
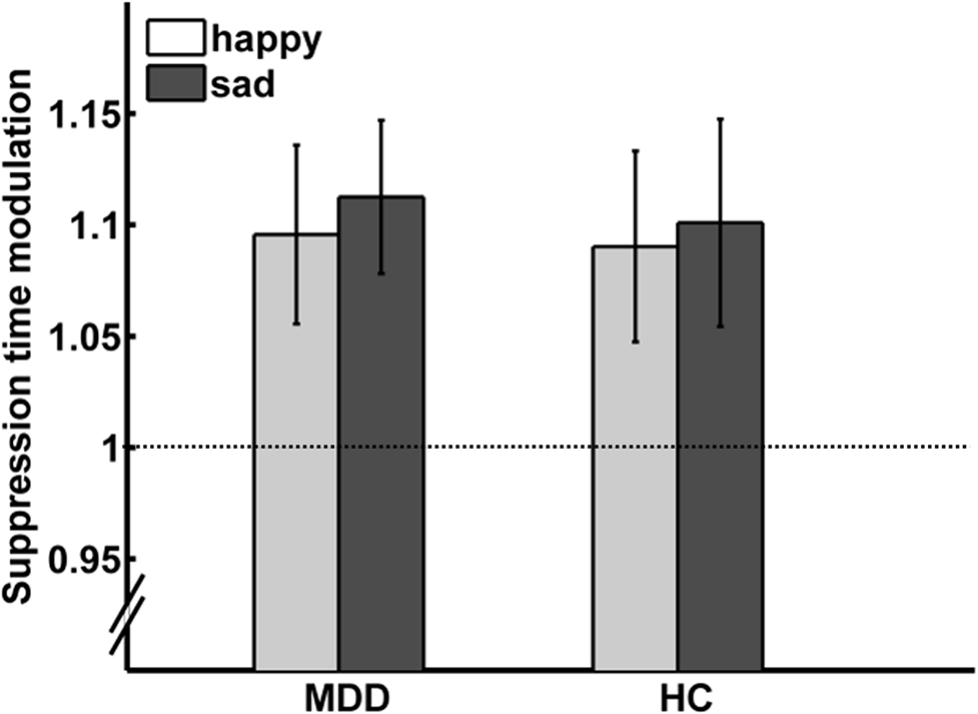
Suppression time modulation by sad and happy faces at T1. Bar plots depict suppression time modulations for happy and sad facial expressions relative to neutral expressions for patients with major depressive disorder (MDD) and healthy participants (HC) at T1. Values > 1, represented by the dotted line, indicate longer suppression times in relation to neutral faces. No differences between emotions or groups were found. Error bars indicate within-subject standard-errors of the mean [53] MDD = major depressive disorder; HC = healthy controls.

After ruling out differences between groups in overall suppression times (t(52) = 1.318; p = 0.193), overall reaction times (t(52) = 0.796; p = 0.430) and suppression times for the neutral expression (t(52) = 1.152; p = 0.169) on the basis of two-sample t-tests, we compared suppression time modulation [19]. Modulation of suppression time (suppression time for emotional divided by suppression time for neutral faces) for both groups at T1 is depicted in Fig 5. A 2 x 2 repeated measures ANOVA with the between-subject factor group and the within-subject factor emotion yielded no significant main effect of emotion (F(1,52) = 0.076; p = 0.784), no main effect of group (F(1,52) = 0.079; p = 0.780) and no emotion x group interaction (F(1,52) = 0.004; p = 0.951).

### Perceptual bias and access to awareness

We investigated whether the perceptual bias observed in Experiment 1 is related to automatic processing of emotional expressions as assessed with CFS in Experiment 2 in patients with MDD. Since differences between groups in Experiment 1 were only found for the PSEs of the logistic functions, we only included the PSEs in this analysis as a measure for perceptual bias. We performed a Pearson correlation between perceptual biases assessed in Experiment 1 and access to awareness assessed in Experiment 2, which was computed as the difference between the suppression time modulations of happy versus sad faces. However, there was no significant correlation between these two measures of emotion recognition (r = - 0.06 p = 0.769).

## Discussion

We showed a negative bias in recognition of facial affect in patients with a current depressive episode. In a forced choice task, the criterion for the distinction of sad and happy expressions was shifted towards sad in patients with MDD relative to the control group. Patients with MDD classified morphed facial expressions with neutral or near-neutral emotional expression more frequently as sad. For the categorical shift from the perception of sad to happy to occur, patients needed higher intensities of happiness expressed by the face, compared to healthy control participants. After three months, we observed the opposite pattern with now a reduction of the previously observed perceptual bias in MDD patients and an even slightly greater bias towards sad faces in the control group. A reduction in perceptual bias correlated with the reduction of depressive symptoms. In the present study we found no evidence for preferential access to awareness of negative information.

Our results at T1 are in accordance with a number of previous studies that also reported a negative perceptual bias in patients with MDD [2,5,7]. A time- and severity-dependent reduction of this negative perceptual bias in response to subtle emotional face expressions has, in contrast, not been observed previously. In the present experiments we used photographs of faces, as opposed to schematic faces [4,14,38–41]. While schematic faces have the advantage of allowing for a reliable comparison between studies, photographs of faces enable more subtle graduations of expression. We exploited this advantage by using finer modulations of emotional expression, especially in the near-neutral range, than several previous studies [2,5,7,13]. Another possibility to present varying intensity levels of emotional expressions would have been the use of dynamic facial expressions [8], which are easier to detect due to motion signals [42]. The naturalness of a dynamic expression, however, depends on its rate of change over time, which differs between emotions [43]. Thus, different emotions would have to be presented at a different rate of change to keep the level of naturalness constant across emotions. We aimed to avoid this problem, and also potentially confounding effects of recognition or response time differences between groups. Therefore, and based on evidence which suggests that the dynamic component of human faces does not play a decisive role in the recognition of the emotions [44], we decided that the presentation of morphed static in the context of a forced-choice task would be best suited for the purpose of our study.

### Negative perceptional bias is related to depressive state

Regarding the question whether a negative perceptual bias reflects a state or trait marker of depression has been addressed in several previous studies, which yielded heterogeneous results [12–15,45]. However, in these studies emotion recognition during the depressive episodes was not compared to emotion recognition in a later phase within the same individuals. It is possible that reductions of perceptual bias reflect differences between individuals rather than a reduction of perceptual biases over time. In the present study, we employed a repeated-measures design, which can be regarded an optimal design to study the temporal stability of such a negative perceptual bias in depressive patients. We found a reduction of perceptual bias in depressive patients from the initial test session to the following test session three months later. Furthermore, this reduction of an emotion-related negative bias was related to a reduction of depressive symptoms. Thus, to the best of our knowledge, we here show for the first time that a negative perceptual bias in emotion recognition changes with clinical improvement. It can thus be concluded that the change in perceptual bias reflects a change in clinical state rather than a trait marker of depression.

Of note, the significant time-by-group interaction was not only driven by a reduction of perceptual bias in MDD patients, but also by a slight increase in the control group. The interpretation of the latter finding must currently remain speculative. Possibly, the repetition of the task may have had differential effects on patients and control participants, respectively. It has indeed been shown before that sub-clinical mood changes, as elicited for instance by mood inductions, affect observers’ accuracy in emotion recognition [46,47]. However, since we did not assess participants’ current state of mood in the situation of testing, we cannot make any strong conclusions regarding the possible influence of short-term mood fluctuations on participants’ responses. Interestingly, the repeated presentation of faces with varying intensities of emotional expressions influences healthy observers’ sensitivity to the evaluation of facial expressions [48]. It remains a topic for future investigations to what extent such repetition-related changes in the recognition of emotional expressions are related to observers’ changes in mood. Importantly, our data clearly show that repeated presentation of emotional faces had a different impact on patients compared to healthy participants. We can thus rule out that the effect observed in the patient group is a mere artifact of stimulus repetition. Moreover, by assessing current depressive symptoms both at T1 and T2 in patients as well as healthy controls we could rule out the possibility that the change in emotion recognition in the control group was related to a change in mood state in the sense of the occurrence of a depressive episode in formerly healthy control participants.

### Access to awareness

In contrast to our previous results [19] there was no difference in suppression times for happy versus sad facial expressions in the present study. The results presented in [19] concur with several other studies that reported evidence for automatic biases in emotion processing in MDD [41,49,50]. Patients with MDD have been suggested to preferentially attend to negative stimuli [41,51,52]. It seems more likely that the failure to detect a group difference in access of emotional stimuli to awareness in the present study is related to other factors. In addition to differences in sample characteristics that are beyond the influence of the experimenter, the discrepancy between the present results and the earlier findings could be due to differences in study design. In the previous study, fearful faces were included, which might have had an indirect effect on the processing of other emotional expressions. Another possibly more important difference was that the dynamic Mondrian patterns were gradually faded out in the current study, similar to previous work [29], but in contrast to our earlier study [19], in which mask contrast was kept constant. With a constant mask contrast, suppression time is only determined by the properties of the target stimulus (in addition to endogenous factors, such as a depressive episode), while a gradual fade-out of the mask will inevitably lead to a break-through of the target once the mask contrast falls below a critical threshold. This could result in a substantially reduced sensitivity to detect suppression time effects caused either by target stimulus differences or by inter-individual differences that potentially affect access to awareness. While this explanation is speculative at the current stage, future studies should clarify how changes in mask contrast and other variables related to CFS task design influence sensitivity for the detection of intra- and inter-individual differences in access of visual stimuli to awareness.

## Conclusions

This study shows an association of clinical symptoms of depression and a negative cognitive bias in emotion recognition. This finding contributes to our understanding of depressive symptomatology as it shows a clear relationship between current clinical state and emotion perception, suggesting that perceptual biases may play an important role in the pathophysiology of depression. Our findings, while in the context of the current study only meaningful at the group level, may help the future development of tools for the objective assessment of treatment response that may even aid the prognostic evaluation of patients with MDD.

## References

[1] Beck AT. Cognitive Therapy and the Emotional Disorders. Penguin; 1979.

[2] Gollan JK, Pane H, McCloskey M, Coccaro EF. Identifying differences in biased affective information processing in major depression. Psychiatry Res. 2008;159: 18–24. 10.1016/j.psychres.2007.06.011 18342954 PMC2571942

[3] Gur RC, Erwin RJ, Gur RE, Zwil AS, Heimberg C, Kraemer HC. Facial emotion discrimination: II. Behavioral findings in depression. Psychiatry Res. 1992;42: 241–251. 1496056 10.1016/0165-1781(92)90116-k

[4] Hale WW 3rd, Jansen JH, Bouhuys AL, van den Hoofdakker RH. The judgement of facial expressions by depressed patients, their partners and controls. J Affect Disord. 1998;47: 63–70. 9476745 10.1016/s0165-0327(97)00112-2

[5] Liu W, Huang J, Wang L, Gong Q, Chan RCK. Facial perception bias in patients with major depression. Psychiatry Res. 2012;197: 217–220. 10.1016/j.psychres.2011.09.021 22357354

[6] Rubinow DR, Post RM. Impaired recognition of affect in facial expression in depressed patients. Biol Psychiatry. 1992;31: 947–953. 1637932 10.1016/0006-3223(92)90120-o

[7] Surguladze SA, Young AW, Senior C, Brébion G, Travis MJ, Phillips ML. Recognition accuracy and response bias to happy and sad facial expressions in patients with major depression. Neuropsychology. 2004;18: 212–218. 10.1037/0894-4105.18.2.212 15099143

[8] Joormann J, Gilbert K, Gotlib IH. Emotion identification in girls at high risk for depression. J Child Psychol Psychiatry. 2010;51: 575–582. 10.1111/j.1469-7610.2009.02175.x 19788553 PMC2862827

[9] Lopez-Duran NL, Kuhlman KR, George C, Kovacs M. Facial emotion expression recognition by children at familial risk for depression: high-risk boys are oversensitive to sadness. J Child Psychol Psychiatry. 2013;54: 565–574. 10.1111/jcpp.12005 23106941 PMC4063303

[10] Venn HR, Watson S, Gallagher P, Young AH. Facial expression perception: an objective outcome measure for treatment studies in mood disorders? Int J Neuropsychopharmacol Off Sci J Coll Int Neuropsychopharmacol CINP. 2006;9: 229–245. 10.1017/S1461145705006012 16316484

[11] LeMoult J, Joormann J, Sherdell L, Wright Y, Gotlib IH. Identification of Emotional Facial Expressions Following Recovery From Depression. J Abnorm Psychol. 2009;118: 828–833. 10.1037/a0016944 19899852 PMC2837802

[12] Leppänen JM, Milders M, Bell JS, Terriere E, Hietanen JK. Depression biases the recognition of emotionally neutral faces. Psychiatry Res. 2004;128: 123–133. 10.1016/j.psychres.2004.05.020 15488955

[13] Anderson E, Siegel EH, Bliss-Moreau E, Barrett LF. The Visual Impact of Gossip. Science. 2011;332: 1446–1448. 10.1126/science.1201574 21596956 PMC3141574

[14] Levkovitz Y, Lamy D, Ternochiano P, Treves I, Fennig S. Perception of dyadic relationship and emotional states in patients with affective disorder. J Affect Disord. 2003;75: 19–28. 12781346 10.1016/s0165-0327(02)00024-1

[15] Mikhailova ES, Vladimirova TV, Iznak AF, Tsusulkovskaya EJ, Sushko NV. Abnormal recognition of facial expression of emotions in depressed patients with major depression disorder and schizotypal personality disorder. Biol Psychiatry. 1996;40: 697–705. 10.1016/0006-3223(96)00032-7 8894061

[16] Naudin M, Carl T, Surguladze S, Guillen C, Gaillard P, Belzung C, et al Perceptive Biases in Major Depressive Episode. PLoS ONE. 2014;9: e86832 10.1371/journal.pone.0086832 24558363 PMC3928096

[17] Etcoff NL, Magee JJ. Categorical perception of facial expressions. Cognition. 1992;44: 227–240. 1424493 10.1016/0010-0277(92)90002-y

[18] Leppänen JM. Emotional information processing in mood disorders: a review of behavioral and neuroimaging findings. Curr Opin Psychiatry. 2006;19: 34–39. 10.1097/01.yco.0000191500.46411.00 16612176

[19] Sterzer P, Hilgenfeldt T, Freudenberg P, Bermpohl F, Adli M. Access of Emotional Information to Visual Awareness in Patients with Major Depressive Disorder. Psychol Med. 2011;41: 1615–1624. 10.1017/S0033291710002540 21208495

[20] Hamilton M. A rating scale for depression. J Neurol Neurosurg Psychiatry. 1960;23: 56–62. 14399272 10.1136/jnnp.23.1.56 PMC495331

[21] Beck AT, Ward CH, Mendelson M, Mock J, Erbaugh J. An inventory for measuring depression. Arch Gen Psychiatry. 1961;4: 561–571. 13688369 10.1001/archpsyc.1961.01710120031004

[22] First MB, Spitzer RL, Gibbon M, Williams JBW. Structured Clinical Interview for DSM-IV® Axis I Disorders (SCID-I), Clinician Version, Administration Booklet. American Psychiatric Pub; 2012.

[23] Schmidt K, Metzler P. Wortschatztest Göttingen. Hogrefe Verlag; 1992

[24] Oldfield RC. The assessment and analysis of handedness: the Edinburgh inventory. Neuropsychologia. 1971;9: 97–113. 5146491 10.1016/0028-3932(71)90067-4

[25] Gollan JK, McCloskey M, Hoxha D, Coccaro EF. How do depressed and healthy adults interpret nuanced facial expressions? J Abnorm Psychol. 2010;119: 804–810. 10.1037/a0020234 20939654 PMC3805828

[26] Yang Z, Zhao J, Jiang Y, Li C, Wang J, Weng X, et al Altered Negative Unconscious Processing in Major Depressive Disorder: An Exploratory Neuropsychological Study. PLoS One. 6 10.1371/journal.pone.0021881 PMC3130746 21755006

[27] Sterzer P, Haynes J-D, Rees G. Fine-scale activity patterns in high-level visual areas encode the category of invisible objects. J Vis. 2008;8: 10 10.1167/8.15.10 19146294

[28] Sterzer P, Jalkanen L, Rees G. Electromagnetic responses to invisible face stimuli during binocular suppression. NeuroImage. 2009;46: 803–808. 10.1016/j.neuroimage.2009.02.046 19285140

[29] Yang E, Zald DH, Blake R. Fearful expressions gain preferential access to awareness during continuous flash suppression. Emotion. 2007;7: 882–886. 10.1037/1528-3542.7.4.882 18039058 PMC4038625

[30] Pollak SD, Kistler DJ. Early experience is associated with the development of categorical representations for facial expressions of emotion. Proc Natl Acad Sci U S A. 2002;99: 9072–9076. 10.1073/pnas.142165999 12072570 PMC124425

[31] Jaeger TF. Categorical Data Analysis: Away from ANOVAs (transformation or not) and towards Logit Mixed Models. J Mem Lang. 2008;59: 434–446. 10.1016/j.jml.2007.11.007 19884961 PMC2613284

[32] Gueorguieva R, Krystal JH. Move over ANOVA: progress in analyzing repeated-measures data and its reflection in papers published in the Archives of General Psychiatry. Arch Gen Psychiatry. 2004;61: 310–317. 10.1001/archpsyc.61.3.310 14993119

[33] Moscatelli A, Mezzetti M, Lacquaniti F. Modeling psychophysical data at the population-level: the generalized linear mixed model. J Vis. 2012;12 10.1167/12.11.26 23104819

[34] Bates D, Mächler M, Bolker B, Walker S. Fitting linear mixed-effects models using lme4. ArXiv Prepr ArXiv14065823. 2014; Available: http://arxiv.org/abs/1406.5823

[35] Tukey J. Exploratory data analysis. Addison-Wesley;

[36] Faul F, Erdfelder E, Lang A-G, Buchner A. G*Power 3: A flexible statistical power analysis program for the social, behavioral, and biomedical sciences. Behav Res Methods. 2007;39: 175–191. 10.3758/BF03193146 17695343

[37] Cohen J. Statistical Power Analysis. Curr Dir Psychol Sci. 1992;1: 98–101.

[38] Bouhuys AL, Geerts E, Gordijn MC. Depressed patients’ perceptions of facial emotions in depressed and remitted states are associated with relapse: a longitudinal study. J Nerv Ment Dis. 1999;187: 595–602. 10535652 10.1097/00005053-199910000-00002

[39] Bouhuys AL, Geerts E, Mersch PP. Relationship between perception of facial emotions and anxiety in clinical depression: does anxiety-related perception predict persistence of depression? J Affect Disord. 1997;43: 213–223. 9186792 10.1016/s0165-0327(97)01432-8

[40] Hale WW 3rd. Judgment of facial expressions and depression persistence. Psychiatry Res. 1998;80: 265–274. 9796942 10.1016/s0165-1781(98)00070-5

[41] Suslow T, Junghanns K, Arolt V. Detection of facial expressions of emotions in depression. Percept Mot Skills. 2001;92: 857–868. 11453215 10.2466/pms.2001.92.3.857

[42] Ceccarini F, Caudek C. Anger superiority effect: The importance of dynamic emotional facial expressions. Vis Cogn. 2013;21: 498–540. 10.1080/13506285.2013.807901

[43] Sato W, Yoshikawa S. BRIEF REPORT The dynamic aspects of emotional facial expressions. Cogn Emot. 2004;18: 701–710. 10.1080/02699930341000176

[44] Gold JM, Barker JD, Barr S, Bittner JL, Bromfield WD, Chu N, et al The efficiency of dynamic and static facial expression recognition. J Vis. 2013;13 10.1167/13.5.23 PMC3666543 23620533

[45] Lewinsohn PM, Steinmetz JL, Larson DW, Franklin J. Depression-related cognitions: antecedent or consequence? J Abnorm Psychol. 1981;90: 213–219. 7288016 10.1037//0021-843x.90.3.213

[46] Hills PJ, Werno MA, Lewis MB. Sad people are more accurate at face recognition than happy people. Conscious Cogn. 2011;20: 1502–1517. 10.1016/j.concog.2011.07.002 21813288

[47] Lee TMC, Ng EHH, Tang SW, Chan CCH. Effects of sad mood on facial emotion recognition in Chinese people. Psychiatry Res. 2008;159: 37–43. 10.1016/j.psychres.2007.04.022 18329723

[48] Moriya J, Tanno Y, Sugiura Y. Repeated short presentations of morphed facial expressions change recognition and evaluation of facial expressions. Psychol Res. 2013;77: 698–707. 10.1007/s00426-012-0463-7 23179582

[49] Dannlowski U, Ohrmann P, Bauer J, Kugel H, Arolt V, Heindel W, et al Amygdala reactivity predicts automatic negative evaluations for facial emotions. Psychiatry Res. 2007;154: 13–20. 10.1016/j.pscychresns.2006.05.005 17182226

[50] Victor TA, Furey ML, Fromm SJ, Öhman A, Drevets WC. Changes in the neural correlates of implicit emotional face processing during antidepressant treatment in major depressive disorder. Int J Neuropsychopharmacol. 2013;FirstView: 1–14. 10.1017/S146114571300062X 23809145

[51] Gotlib IH, Krasnoperova E, Yue DN, Joormann J. Attentional biases for negative interpersonal stimuli in clinical depression. J Abnorm Psychol. 2004;113: 121–135. 10.1037/0021-843X.113.1.121 14992665

[52] Gotlib IH, Kasch KL, Traill S, Joormann J, Arnow BA, Johnson SL. Coherence and specificity of information-processing biases in depression and social phobia. J Abnorm Psychol. 2004;113: 386–398. 10.1037/0021-843X.113.3.386 15311984

[53] Cousineau D. Confidence intervals in within-subject designs: A simpler solution to Loftus and Masson’s method. Tutor Quant Methods Psychol. 2005; Vol. 1(1): 42–45.

